# Reducing the Consumer Attitude–Behaviour Gap in Animal Welfare: The Potential Role of ‘Nudges’

**DOI:** 10.3390/ani8120232

**Published:** 2018-12-05

**Authors:** Belinda Vigors

**Affiliations:** Scotland’s Rural College (SRUC), West Mains Road, Edinburgh EH9 3RG, UK; belinda.vigors@sruc.ac.uk

**Keywords:** animal welfare, behaviour change, behavioural economics, nudge, choice architecture, social norms, pre-commitments

## Abstract

**Simple Summary:**

Many members of the public express a desire for farm animals to have a good quality of life. Yet, when it comes to purchasing higher welfare products which would support this, many consumers do not ‘walk their talk’. This paper introduces the concept of ‘nudging’ as a means to help consumers align their actions with their intentions and support their desire to engage in pro-animal welfare behaviours. ‘Nudging’ is a collection of behaviour change tools designed to hint to, or suggest, a choice most closely aligned with an individual’s self-interests or intentions. Their purpose is to simplify the decision-making environment by working in concert with the behavioural flaws known to influence human decision-making. Four specific behavioural ‘nudges’ are outlined: self-nudges, choice architecture, social norms and pre-commitments, along with examples of how they can be applied to animal welfare. Inspired by effective applications of ‘nudging’ to close the consumer attitude–behaviour gap in other relevant domains, this paper seeks to highlight how similar initiatives might be applied to better support higher welfare choices amongst consumers and in turn, enhance the lives of farm animals.

**Abstract:**

Citizen concern for the welfare of farm animals is well documented. However, there is a notable gap between people saying they want improved farm animal welfare and how they actually behave as a consumer. This is known as the citizen–consumer attitude–behaviour gap. As improvements in farm animal welfare can be affected by market demand, the choices consumers make become important. This paper introduces the concept of ‘nudging’ and discusses how it could be applied to reduce the attitude–behaviour gap amongst consumers. By designing the choice environment to better reflect the behavioural biases known to impact human decision-making, ‘nudge’ tools function to prompt individuals to make choices that are aligned with their stated intentions. Four ‘nudge’ tools: self-nudges, choice architecture, social norms and pre-commitments are discussed. The behavioural rationales for their use are reviewed and examples of how they might be applied to animal welfare provided. Improved farm animal welfare arguably requires improved pro-welfare consumer behaviour. This paper highlights how this might be encouraged by: self-nudging the salience of an ethical self-image; altering the choice architecture to influence decision-making; articulating social norms to impact behaviour; and using pre-commitment devices to overcome self-control issues.

## 1. Introduction

Increasing public concern for the welfare of farm animals is well documented [1,2,3]. Yet, this growing interest in the lives of farm animals does not correspond with an increase in demand for higher welfare products [4]. This phenomenon—better known as the citizen–consumer attitude–behaviour gap—occurs when individual citizens who express an interest in farm animal welfare make consumer purchase decisions which do not reflect this. In other words, “animal welfare-minded consumers do not always walk their talk” [5] (p. 1683). This is particularly relevant considering the increasingly market-driven nature of animal welfare, where improvements to welfare above minimum standards can be driven by consumer demand and supported by consumer purchase of higher welfare products sold at premium prices [6]. Addressing this attitude–behaviour gap and supporting consumers to make higher animal welfare choices thus plays an important role in enhancing the lives of farm animals.

‘Nudging’, a behaviour change tool of behavioural economics, is now a well-recognised and effective policy tool to align individual intention and action [7]. A ‘nudge’ is an intervention which seeks to influence peoples’ decisions or behaviours in a predictable way—often by changing the conditions of the decision-making environment—with the goal of aligning an individual’s behaviour with their self-stated interests [8]. Fueled by numerous successful applications [9], ‘nudging’ is increasingly used to deal with similar citizen attitude–behaviour gaps in policy domains such as health and the environment [7,10,11]. For example, changing the layout of cafeterias so that healthy foods are in noticeable areas and unhealthy foods are in less noticeable areas has helped ’nudge’ people to make healthier food choices [9]. Similarly, placing fruit next to check-outs in supermarkets can ‘nudge’ people to buy healthy foods by making these salient at the point of purchase [12]. Furthermore, colour-coding recycling bins can ‘nudge’ people to sort their recyclables properly, by making it easier and quicker to determine the correct collection bin [13].

When it comes to animal welfare, consumers are offered an array of ‘animal-friendly’ food choices (e.g., free range, high welfare assured, organic) and can access numerous information sources to educate themselves on how to make ‘animal-friendly’ purchases [1,14]. However, if it was not already difficult enough to make a decision when such choices often overlap with other attributes (e.g., health, quality, taste) and educational messages about welfare are not always perceived as reliable [15], the very nature of human decision-making further constrains pro-animal welfare behaviours in this context [16]. For instance, rather than effortfully weighing up all the options or seeking out additional information, we humans consistently rely on ‘rule-of-thumb’ thinking or ‘heuristics’ when making decisions [17,18]. Thus, the effectiveness of education and greater product choice is constrained by our analytical limitations. Additionally, many of our decisions are influenced by how information is presented, the cognitive reference points we draw from and our past experiences (amongst others) [19]. Thus, the way in which animal-welfare choice options are presented and the nature of the decision context may affect consumer decision. Furthermore, we often deviate from long-term goals and make decisions which do not serve our own interests [20] or reflect what we believe is the ‘right’ thing to do [18]. As such, even with the best of intentions, when it comes to the moment of decision it can be very difficult for an individual to make an ‘animal-friendly’ consumer choice; hence the attitude–behaviour gap [16]. Nudges are argued to help overcome attitude–behaviour gaps because they work in concert with the limitations of human decision-making and the, often unconscious, factors driving behaviour [7,9,21]. As such, nudging may prove to be an effective behaviour change tool to encourage greater ‘animal-friendly’ action amongst consumers who express such intentions.

The aim of this paper is to highlight the potential of ‘nudging’ to align citizen intention and consumer action in the context of farm animal welfare (i.e., reduce the attitude–behaviour gap). The ‘nudge’ literature would suggest that if citizens are expressing pro-animal welfare intentions but not pro-animal welfare behaviours [4], then aspects of the consumer choice environment may be negatively affecting their decision-making [9]. Nudging offers a means to better support consumers to make choices aligned with their intentions, by altering the consumer choice environment in a way that takes account of the behavioural biases affecting human decision-making [16]. As such, the first objective of this paper is to introduce to those working in the field of farm animal welfare the central tenets of behavioural economics—the field in which ‘nudging’ originated—and its implications for the design of behaviour change initiatives. Secondly, it seeks to highlight four key ‘nudges’ which could be applied to encourage ‘animal-friendly’ choices amongst consumers. Namely: self-nudges, choice architecture, social norms, and pre-commitments. This paper then reviews applications of these nudges in policy domains where similar attitude–behaviour gaps are found (e.g., health and environment) and suggests how this knowledge might be applied to better align consumer behaviour with their pro-animal welfare intentions. In turn, it is hoped that this paper will spark discussion and further debate of how consumer choice of higher welfare products can be encouraged and better supported along these lines.

## 2. A Brief Introduction to Behavioural Economics and ‘Nudging’

To understand the concept of ‘nudging’ and how such tools are proposed to work; it is beneficial to explore its theoretical origins in the field of behavioural economics.

Behavioural economics developed from a recognition that the behaviour of individuals often deviates from the neoclassical economics’ characterisation of people as rational actors [22]. One of the central predictions of this ‘rational’ characterisation is that an individual’s decisions are largely driven by self-interest or what provides the greatest satisfaction (i.e., motivated by a need to maximise utility) [23]. However, behavioural economics questions this as many of the decisions we make are influenced by our social norms; the implicit rules or behavioural expectations within a group, such as a sense of fairness or reciprocity [24], rather than our own self-interests [25]. Indeed, within animal welfare, Napolitano et al. [26] notes that citizen desire to express a pro-animal welfare attitude may result from trying to conform with social norms. Similarly, Norwood and Lusk [27] argue that consumers participating in animal welfare research may exhibit social desirability bias, where they seek to display what they believe is a socially acceptable response (e.g., ‘I value animal welfare’). Thus, individual consumers in this domain appear more affected by social expectations than self-interested desires.

A further prediction of neoclassical economics is that an individual’s decisions are made according to a consistent set of stable preferences (i.e., they do not change between or within decision contexts) [23]. However, research in behavioural economics has demonstrated that our preferences are rarely stable but vary between and within decision contexts, often because of how information is presented to us [28,29]. For example, Fox et al. [30] found that a positive description of food irradiation increased consumer willingness-to-pay for a pork sandwich, while a negative description decreased willingness-to-pay for the same product. Indeed, the citizen–consumer attitude–behaviour gap demonstrates a deviation from the neoclassical assumption of stable preferences, as the citizen’s pro-animal welfare attitude does not hold in a stable way when they enter the consumer context.

An additional issue with the neoclassical economic framework is that it predicts that a ‘rational’ individual will seek out further or missing information if they do not already have enough to make an appropriate decision [23]. However, it is now well-recognised that we humans are bounded in our rationality; our ability to seek out further information is limited by the time we have and the information available, while our own analytical power to analyse lots of information and choose between options is constrained [31,32]. Such bounded rationality has implications for animal welfare. For instance, increased information provision (e.g., through education) is proposed as a way to increase pro-animal welfare behaviours amongst consumers [33]. Yet as Ingenbleek et al. [34] (p. 817) note, “people do not always make the time to consider animal welfare specifically when making their purchase decisions”. In other words, consumers are bounded in the time to seek out additional information to make a decision. Moreover, some consumers do not possess a desire to be educated but actively choose to remain uninformed or ‘willfully ignorant’ [35]. For instance, Bell et al. [35] found that a third of survey participants (in a sample of 1000) chose to look at a blank screen rather than see an informative image about how pregnant sows are housed. In addition to education, providing greater choice in the offering of higher welfare products is proposed as a means to harvest greater willingness-to-pay from the market through increased product differentiation [34]. However, the seminal work of Iyengar and Lepper [36] demonstrated that when a consumer has too many choices they are less likely to buy; too much choice overloads a person’s cognitive analytical ability and constrains (i.e., ‘bounds’) their decision-making. Indeed, Iyengar and Lepper [36] found that consumers were more likely to make a purchase (in this case; jam) when presented with a limited offering of six choices than when presented with an extensive offering of 24 choices.

The issue, from a policy design or behaviour change point of view, is that the neoclassical economic framework has continued to influence models and predictions of human behaviour, despite awareness of the limits of its predictive power [19]. The relevance of behavioural economics here is that it takes account of, what the neoclassical economic framework would dismiss as, irrational behaviours and uses them to improve the behavioural realism of economic models. It is this behavioural realism which has led to the increasing appeal and prominent application of behavioural economics to improve evidence-based policy making [37]. There are now nearly 150 governments worldwide making use of behaviourally informed ‘nudge’ tools [38] such as “warnings, reminders, social norms and default rules” [39] (p. 3) to influence citizen behaviour and choice. 

‘Nudging’ is a term which refers to a collection of behavioural economics tools (for a comprehensive list see Sunstein [40]) which influence behaviour by accounting for the nuances of human decision-making. As Hausman and Welch [41] (p. 126) explain:

“Nudges are ways of influencing choice without limiting the choice set or making alternatives appreciably more costly in terms of time, trouble, social sanctions and so forth. They are called for because of flaws in individual decision-making, and they work by making use of those flaws”.

In other words, nudges are tools which influence behaviour by harnessing behavioural economics’ understanding of what drives human behaviour (i.e., its irrationalities, flaws or biases). One of the key ways in which nudges do this is by changing the ‘choice architecture’ of a decision context. This involves reshaping the environment in which a decision is made, usually by changing the way in which choice options are presented. The logic for this is derived from the previously outlined tenets of behavioural economics; that an individual’s choices are influenced by behavioural biases such as how information is presented, the information they have available and the social norms or rules made salient by the context (amongst others). As such, by adhering to the innate features of human decision-making, nudges function to make decisions easier and help individuals behave in their own self-interests without compromising their freedom of choice (as bans, penalties and other harder interventions do) [25]. The analogy often used is of a GPS system; it makes deciding what route to take clear and simple but the individual does not have to follow it if they do not want to [40].

Importantly, nudging does not assume that people “lack knowledge or information, [but] that they lack the *ability* to process all of the complex and multiple information sources being thrown at them” [42] (p. 5). Nudging takes account of the fact that people often make decisions which are not in their best interests because humans are heavily influenced by the way in which choices are presented, are constrained in self-control and analytical capacity, and generally rely on mental shortcuts when making decisions [12]. This is particularly relevant to the animal welfare attitude–behaviour gap where, as previously mentioned, many citizens express a desire to make ‘animal-friendly’ consumer choices but may be constrained in doing so by the current choice environment. As Norwood and Lusk [43] (p. 265) point out, “the incentives in the grocery store are different”, meaning consumer choice can be affected by external factors (e.g., supermarket lay-out, presentation of information etc.) which may cause them to deviate from their pro-animal welfare intentions. As such, nudging argues that deliberate changes to the choice environment can help people make choices that are in their better interests. For animal welfare, this would involve using nudges to more closely align an individual’s citizen intention with their consumer action.

The relative success achieved by bringing such behavioural insights into policy-making has challenged and led many to rethink the “traditional view that behaviour change should be achieved by informing, convincing, incentivising or penalising people” [9] (p. 4). This is of note for animal welfare where education, incentivisation and legislation are common policy strategies to encourage higher welfare choices [6,33,44]. As animal welfare in many countries is increasingly market-driven, further improvements to the lives of farm animals may be affected by consumer demand for higher welfare products [44,45]. With this in mind, application of the behavioural insights provided by behavioural economics has the potential to improve consumer engagement with animal welfare, particularly as nudges are less administratively burdensome or costly than traditional behavioural interventions [46]. The following section sets out how ‘nudging’ might help reduce the consumer attiude–behaviour gap by presenting four hypothetical examples of potential nudge tools; (1) self-nudges; (2) choice architecture; (3) social norms and; (4) pre-commitments. The key point is that “even when people are motivated to make personally and socially desirable choices, external constraints in the decision process or aspects of the decision task can prevent them from choosing optimally” [16] (p. 385). As such, the aim of each of the following ‘nudges’ is to make it easier for a citizen with a pro-animal welfare attitude to action this when in a consumer context, by setting up the decision-making environment to work congruously with the behavioral biases which influence decisions.

## 3. Behavioural Initiatives to ‘Nudge’ Pro-Animal Welfare Behaviour

The main attractions for using nudges is that they are; (a) not coercive and; (b) they are more cost-effective, relative to other more administratively burdensome policies (such as legislation) [7]. The former point is important when considering the heterogeneity of consumers. The presence of a nudge does not result in a specific cost or loss to the individual, rather it is there to make a self-desired choice easier [47]. If some citizens, as research has shown [3,4,14], express a desire to improve the lives of farm animals, then the purpose of nudging is to make that desired intention easier to fulfill and action where it matters (i.e., when they are making decisions as a consumer). For those citizens who have absolutely no desire to engage in pro-animal welfare behaviours, then the nudge should not constrain their free-choice. Furthermore, the variety of nudge tools available means different nudges can be used to support the variety of consumers ranging from highly engaged to those who express more moderate pro-animal welfare preferences. From the hypothetical examples set-out in this section; self-nudges are most suited to highly engaged consumers who actively desire to improve their pro-animal welfare behaviours; choice architecture is suited to influence those citizens who want to support animal welfare but are less motivated to or are constrained (e.g., by time, hyper-choice contexts etc.) in their ability to action this; social norms have the potential to influence those who are less engaged through their effect on the unconscious social factors which affect behaviour and; pre-commitments, similar to self-nudges, are best suited to citizens who have a strong motivation to action their pro-animal welfare intentions in their consumer decisions. A descriptive summary of each of these ‘nudges’, a hypothetical example of how they could be applied to animal welfare, and the behavioural mechanisms they harness to influence consumer decision, is included in Table 1.

### 3.1. Self-Nudges

As noted, this paper is motivated by the attitude–behaviour gap between citizen intention and consumer action, where the purchase behaviour of the consumer does not always reflect their self-image as a citizen with a moral and ethical interest in animal welfare [5,14,48]. Lades [25] proposes that such deviant consumer purchase behaviours—which are potentially impulsive; influenced by price, product placement, special offers etc.—“can be guided to ethical directions *by making salient certain self-images* which favour ethical consumption” (p. 115). This approach may be particularly relevant to the sub-set of consumers who value their image as ethical consumers and have been found to pay more for foods with specific ethical attributes, such as being more ‘animal-friendly’ [49]. To understand how making ethical self-images salient can be actioned through self-nudges, and consequently improve decisions, it is necessary to consider the behavioural processes underlying the attitude–behaviour gap. 

The dual-personality of citizen–consumer animal welfare behaviour has analogies with the dual process models of human decision making. The most well-known of these is Kahneman’s [50] ‘System 1 and System 2’ model. It describes how our behaviour is driven by two systems; system 1 which is our intuitive, implicit, automatic, associative fast-thinking brain, and system 2; which is our effortful, analytical slower-thinking brain. Lades [25] describes how the System 1 and 2 model can elucidate anomalies in ethics-driven consumer behaviour (i.e., the attitude–behaviour gap). The purpose of system 2 within choice situations, is to set long-term goals and guide the individual towards the achievement of these goals [25]. In the context of animal welfare, this can be a citizen’s moral or ethical intention to buy higher welfare products—it is their salient self-image as a moral, ethical individual who values the lives of farm animals [4]. The purpose of system 1 is to speed up and simplify decisions, often by relying on heuristics and filtering the information presented to us (i.e., noticing what is most salient), hence it is utilised when in the hyper-choice environment of consumer purchase decisions [16,25].

Essentially, a citizen’s self-image as being an ‘ethical animal-welfare oriented’ individual is harboured by the system 2 brain, which guides decisions which are for the long-term good of the individual [25]. However, the system 1 brain tends to deal with short-term, immediate decisions, which may lead to digressions from long-term goals. This dichotomy in the mechanisms of choice becomes important in complex decision contexts, such as supermarkets, where large amounts of information must be processed quickly and with minimal effort. Thus, on entering the supermarket the consumer system 1 brain will take over as it seeks to simplify the array of information presented (e.g., price, special offers, labeling, product placement, quality, nutritional value, visceral effects etc.) and enable fast decision-making (e.g., rely on salient environmental cues). The citizen system 2, ethics-oriented brain is too effortful and slow to deal with this decision context (see Koster [51] for further review of how system 2 fails in food purchase contexts), hence the individual citizen may fail to action their pro-animal welfare intentions as a consumer.

One way to intervene and encourage more pro-animal welfare or ‘ethical’ consumer behaviour would be to change the way in which choice information is presented in the supermarket (e.g., change the choice architecture, as will be presented in the subsequent section). In other words, use an external nudge; the more traditional idea of what ‘nudging’ involves. However, as Lades [25] notes, a self-nudge (i.e., one that is internally generated) may be more powerful as it is designed by the individual to trigger what they consider is in their best interests (i.e., ‘animal-friendly’ purchases) and can be tailored by them to suit their individuality. This approach is likely most relevant to those citizens who express a strong ethical or moral interest in animal welfare and actively want to enhance their pro-animal welfare consumer decisions. Ghvanidze et al. [52] find that consumers engage more with ethical attributes of food when they believe there is something they can do to make a difference (i.e., their actions effect change). By providing motivated citizens with a way to support and action their pro-animal welfare intentions, self-nudges may help reduce the citizen–consumer attitude–behaviour gap seen in animal welfare.

Lades [25] proposes two main ways in which an individual could self-nudge ethical purchase decisions. The first is to reduce their impulsive consumption of unethical products (e.g., lower welfare products), the second is to induce their impulsive consumption of ethical products (e.g., higher welfare products). A potential way of achieving the first is for the individual to avoid situations where “they are exposed to cues that can interact with visceral states and thus trigger impulsive urges to consume goods they consider to be unethical” [25] (p. 123). For example, the individual could avoid mainstream supermarkets, where they might impulsively buy a lower welfare product they consider unethical. Instead, they could frequent local farmers markets, farm shops or use online meat-box delivery services where their self-image as an ethical consumer is salient and thus supports their purchase of higher welfare products. Toma et al.’s [15] study of European consumers provides evidence that some consumers may be willing to do this, finding that access to information on welfare (which may contribute to the greater ethical motivation of the consumer) was the strongest determinant of willingness to change their usual place of shopping to access more animal-friendly products. Moreover, Torma et al. [53] investigated the effectiveness of this self-nudge approach in a qualitative study of consumers’ subscription to an organic grocery delivery service. They found that such consumers consciously made the change from supermarket to grocery box delivery because it reduced the complexity of making many small decisions in the supermarket, to one simpler, larger decision which adhered to their pro-sustainable intentions [53]. As such, they effectively self-nudged themselves to better align their intentions with their behaviours. Importantly, Torma et al. [53] note that consumers who already possessed strong pro-sustainable attitudes achieved higher levels of goal fulfillment by using the organic grocery subscription, while those with lower pro-sustainable attitudes strengthened their attitude toward organic food consumption and some even paid more attention to eco products on normal shopping trips. This suggests that such self-nudges can work by enhancing an individual’s image as an ethical consumer, as suggested by Lades [25]. However, it is not always possible for consumers to avail of such services or avoid mainstream supermarkets.

As such, the second self-nudge strategy where the individual self-nudges impulsive consumption of ethical products becomes important, doing so requires the individual to actively make salient their self-image as an ethical consumer [25], so that their system 1 thinking may intuitively choose the ethical product (e.g., higher welfare). Lades [25] suggests that thinking about past vices before entering a grocery store (e.g., previously not buying the higher welfare product) may be enough to do this. Increasing their consumer knowledge and exposing themselves to key environmental cues such as ‘organic’ or ‘free range’ labels can also help an individual self-nudge ethical consumer behaviour [25]. Indeed, Akaichi et al. [54] found that when consumers began to associate organic bacon with higher welfare attributes, demand for it increased amongst ethical consumers. In addition, the use of shopping lists is known to influence what is purchased and what is avoided in the supermarket and thus may be effective for prompting pro-animal welfare choices [55]. For example, consumers could write ‘high welfare chicken’ rather than just ‘chicken’ on their shopping list so that their ethical self-image is salient as they browse the aisles.

In sum, if a citizen already possesses an ethical or moral interest in animal welfare, utilising self-nudge strategies may be particularly effective; increasing the salience of their ethical self-image may increase the likelihood that they will make pro-animal welfare purchases, as they are less susceptible to associative, affectual system 1 thinking.

### 3.2. Choice Architecture

The self-nudge approach described in the previous section is arguably only applicable to the individual citizens who have a strong moral or ethical conviction towards animal welfare and are motivated to enhance their buying behaviour. Given the heterogeneity of consumers [56], there are likely many who desire to engage in pro-animal welfare purchase choices but are not so highly engaged as to enact self-nudges. This is where altering the choice architecture—the way in which information and choice options are presented to an individual—may prove particularly effective. Such an approach is a well-supported behavioural nudge for influencing choice, particularly in choice situations where individuals often deviate from their intentions (e.g., citizens who express pro-animal welfare intentions but fail to enact this at point of purchase) [9]. Given the market-driven nature of animal welfare, many of the consumer choices impacting farm animal welfare are made in the supermarket. As such, changing supermarket choice architecture to better support pro-animal welfare purchase decisions may be an effective intervention. 

Previous research has demonstrated that factors such as shelf height and lay-out, product placement and aisle design, amongst many others, influence how individuals perceive a supermarket, the products they buy and how much they buy [57,58]. However, for choice architecture to work effectively, it requires a much more deliberate consideration of what decision-making bias is likely to have the strongest influence on the target group. The environment can then be designed accordingly to better support and steer individuals towards their desired choice (e.g., ‘animal-friendly’ products), without limiting choices for others (e.g., those with no animal welfare intentions) [9,47]. Product price is arguably a key aspect of current supermarket choice architecture, as price exists as a key decision-making tool for consumers [59]. Indeed, studies within the animal welfare literature have shown that for many consumers the price of the product is the main choice criterion [4,6,60]. As such, considering how the current price choice architecture in supermarkets operates may reveal why consumers are often swayed from their pro-animal welfare citizen intentions.

In this regard, an interesting finding of the behavioural pricing literature is that it is not the absolute price per se that influences consumer purchases, but the relative price [61]. This means purchase decision-making is affected by how the price of one product ranks compared to another (e.g., is it higher or lower?). Central to understanding this is the concept of reference points; humans rarely make decisions in isolation but evaluate choices relative to a cognitive reference point [62]. As such, when individuals make product choices they have a reference price in mind [63]. This acts as an anchor for price evaluation, consequently influencing consumer perception of price and purchase decisions [64,65]. If relative price triggers such behavioural mechanisms and biases, then it is potentially a worthy target for choice architecture.

This phenomenon, which has become known as the reference price concept, is found to be one of the central drivers of consumer decision-making in price evaluation scenarios [64]. Although reference price is difficult to conceptualise due to its intrapersonal nature, the generally held view is that it has both temporal and contextual components [64,66]. The former being the internal reference price a consumer assimilates from their memory of the highest and lowest prices paid [67] and the latter being the external in-store prices at time of purchase [66]. The external contextual reference price is argued to be more important [63] because the in-store price is the most recent and salient at time of choice [66]. In this regard, Adaval and Monroe [59] argue that reference prices are formed from exposure to stimuli which consumers are not consciously aware of and consequently, guide consumer behaviour automatically. Specifically, they found that consumers judge a product to be less expensive when exposed to a high price context, e.g., a product that costs €100 is judged as less expensive in the context of two others which cost €125 and €150 [59]. Conversely, when exposed to a low-price context, consumers tend to judge prices as more expensive, e.g., if a target product costing €100 is displayed in the same context as others which cost €50 and €75 [59]. As such, the context of the prices and the information this conveys of how they rank relative to others can incidentally determine purchase decisions.

Such factors are important for understanding how higher animal welfare purchase decisions can be negatively impacted by the current choice architecture of supermarkets. In considering the various offerings available in a supermarket in terms of animal welfare; the highest cost products are often those in the category of free range, organic or high welfare farm assurance schemes; the mid-range priced products are those which may be part of a farm assurance scheme which assures minimum welfare standards; and then there are the lower priced, standard products. Importantly, these products are usually grouped together according to product type (e.g., poultry, beef, lamb etc.). Thus, a consumer wishing to purchase, for example a 1.5 kg whole chicken, would proceed to the poultry aisle where offerings may range from £10.43 for an organic, free-range chicken down to £2.52 for a standard chicken of the same weight (prices correct at time of writing for UK, mysupermarket.co.uk, 2018). Due to the influence of reference pricing, the highest priced product may be perceived as too expensive (relative to others) while the lowest price product may be deemed poor quality (relative to others) [68]. As such, the consumer may choose a product near the median which may be meeting minimum but not higher welfare standards.

A key finding of ‘nudging’ and its associated literature is that the choice a person makes depends greatly on how that choice is presented to them [69]. As such, deliberate changes to the choice architecture can nudge people to make better decisions [9]. Johnson et al. [69] advises that choice architecture can focus on; (i) changing what is presented to decision-makers in a choice task (i.e., the structure of the choice); and (ii) altering how choice options are presented (i.e., the description of the choices). Adaval and Monroe’s [59] finding that consumers are unconsciously influenced by whether they are exposed to high or low-price contexts, gives some insight as to how the supermarket choice architecture for higher welfare products could be effectively structured and described. One potential way would be to group products according to welfare provision, whereby a specific ‘higher welfare’ section or aisle could be created and labeled as such, comparable to the ‘free from’ sections seen in many supermarkets (‘Free from’ sections are specific areas where products catering to those with food intolerances such as gluten and dairy, are displayed). Of course, a form of sectioning does already exist in many supermarkets, where the higher welfare or higher cost products tend to be ‘top-shelf’ and organic products sometimes have their own section. However, as van Herpen [57] explains, the specific way in which products are displayed or organised matters, because this determines the salience of product attributes, which in turn influence consumer decision. Thus, to get the greatest benefits from reference pricing, higher welfare products would need to have a clearly defined and delineated section, potentially at the end of an aisle or an aisle of its own so that its product attributes are clearly defined and at their most salient. To support decision-making, the consumer should be able to clearly and quickly pick-out the higher welfare section and, if interested, proceed to it to make a choice.

Doing this would support ‘animal-friendly’ decision-making via several choice architecture mechanisms. Firstly, such sectioning would change what (i.e., structure of the choice) is being presented to decision-makers. Rather than an extensive section (e.g., beef or poultry) with a large variance between highest and lowest prices, consumers would be presented with an exclusive section, with minimal variance between highest and lowest prices. This would provide what Adaval and Monroe [59] term a high-price context, whereby paying £5 for a high welfare product may be perceived as good value relative to other high welfare products priced at £7 and £10, thus minimising the impact of external contextual reference prices on purchasing behaviour.

Secondly, the ‘partitioning’ of options into groups and categories is a well-supported choice architecture intervention which works by changing *how* choice options are presented [69,70]. For instance, Fox et al. [70] found that when they categorised healthy food options on menus into specific, separate categories of ‘fruit’ and ‘vegetables’, and unhealthy food options into a single category of ‘cookies and candies’, consumers purchased more healthy food. Interestingly, Schjøll and Alfnes [71] found that providing descriptions on a menu that veal was organic or had animal-friendly attributes (e.g., ‘from happy calves’) had little effect on consumer choice. In line with behavioural economic tenets, individuals were likely bounded in their ability to analyse such extra information, and hence its limited effect on their behaviour. Indeed, part of the reason proponents of partitioning argue it is effective is because it makes it easier for individuals to choose from the array of options by reducing the need for effortful analysis of information [70]. As such, partitioning food products into categories of ‘higher welfare’ may better support their purchase decisions [72]. Moreover, a particular benefit of partitioning is that it is thought to have the strongest effect amongst decision-makers who are less intrinsically interested in the target subject [69]. Therefore, the partitioning of higher welfare products may be an effective intervention for those consumers who express pro-animal welfare intentions but are not motivated enough to action self-nudges, as described in the previous section. 

Thirdly, providing an animal welfare section supports the description of choices by emphasising the animal-friendly attributes of the products. When making choices, decision-makers often use attribute information to predict how satisfied they would be with different options [69]. Consumers can become overwhelmed when presented with too many attributes, which is often the case with animal welfare as such products are “displayed and sold along with food products carrying other attributes such as organic, local, fair trade, etc.” [5] (p. 117). As such, emphasising a subset of attributes or highlighting one important attribute, in this case animal welfare, reduces the cognitive effort required by the decision-maker and nudges them toward actioning their desired higher animal welfare intentions [69]. For example, in the context of pro-environmental purchases, Theotokis and Manganari [73], found that simplifying the attributes of products down to a ‘green’ and ‘non-green’ option—forcing consumers to choose between the two—was more effective at increasing the purchase of ‘green’ products. Potentially, offering animal-derived food products in a similar way may have a comparable effect.

Although choice architecture can be a powerful tool to help consumers align their actions with their intentions, a caveat of the approach described here is that it requires the willingness of supermarkets to carry it out. The main issue is that the retail margins of food are very narrow and supermarkets are already environments that are ‘busy’ with behavioural tools (e.g., special offers, discount sections, promotions etc.) [74]. However, restaurants face similar issues with thin profit margins on food sales, yet nudging (e.g., menu partitioning as noted above [70]) has proved successful here. Moreover, the choice architecture approach outlined in this section may be effective because it moves the animal welfare interested consumer away from the ‘busy-ness’ of the main meat product section, into a dedicated higher welfare section where reference pricing can have a more positive effect on purchase decisions. As higher welfare products tend to be higher priced, if such sectioning results in an adequate increase in purchases, supermarkets may be persuaded to use this approach. Nonetheless, given the higher price of most higher welfare products, this also raises questions about whether consumers would be ‘nudged’ to spend more than they can afford. It is important to note that sectioning animal welfare products as described should adhere to one of the core principles of nudging; encourage better decisions but not restrict free choice [9,25]. The provision of ‘higher welfare’ sections should have little effect on consumers who have no interest in animal welfare—they can pass by the aisle—but would better support those who have some interest in animal welfare by making decisions easier for them. In other words, a consumer who is interested in animal welfare may be attracted to the higher welfare section and, in the absence of large price differences between the minimum and the maximum, be better supported by the choice environment to make the higher welfare choice they desire to. 

There are numerous ways to alter the choice architecture and those presented here are but a small proportion of them. Although the actual implementation of choice architecture is often straightforward, a constraint on their effectiveness is that it is difficult to determine what or which behavioural bias it should influence [75]. In this section, reference pricing was selected because of the importance many consumers place on price and the potential for it to influence decision at the point-of-purchase. As such, it seemed a most relevant example to illustrate how alterations to supermarket choice architecture could better support higher welfare purchases. Nevertheless, future research to ‘unpack’ which behavioural biases are affecting higher welfare purchases would be most beneficial for the effective design and implementation of choice architecture in this context.

### 3.3. Social Norms

Social norms—the implicit or explicit rules and expectations of a group—automatically guide much of individual behaviour, where what is socially accepted can influence how people behave and respond to a decision context [24,76]. The behavioural impact of social norms has brought into question the neoclassical economics assumption that human behaviour is driven by self-interest [20], motivating many behavioural economists to develop interventions which use social norms to prompt behavioural change in simple and effective ways (see Farrow et al. [76] for a review). In the context of animal welfare, it is often noted that social norms influence and underlie the behaviour of citizens [26,77]. Importantly, social norms are thought to influence behaviour through the fast, automatic, affective mental system and as a consequence, people often do not realise the extent to which they affect their behaviour [78]. As such, social norms could potentially be harnessed to encourage pro-animal welfare behaviours amongst consumers less engaged in animal welfare.

In addition to trying to fit in and avoid social disapproval or conflict, a further influence of social norms is that people look to the behaviour of others to determine what actions are effective and what choices to make [76]. Indeed, when in situations of uncertainty or under cognitive load individuals often use the behaviour of others as a guide for what they should do [76]. Numerous studies, in a variety of contexts, have demonstrated that explicitly telling people what most others do in a given situation (i.e., descriptive social norms) or what others approve of or expect in a given situation (i.e., injunctive social norms), can support behaviour change [79]. Of particular note are social norm interventions in the health and environmental literature, where a consumer attitude–behaviour gap comparable to that found in animal welfare is well documented (e.g., an expressed desire to be ‘green’ does not result in ‘green’ consumer decisions) [18]. For example, exposing students to posters stating: ‘The average student eats three portions of vegetables a day’, was found to increase vegetable consumption amongst students who habitually do not eat vegetables [80]. Similarly, telling people the amount recycled by the average household in their neighbourhood is known to increase the amount and frequency of their recycling [81]. Several studies have also found that towel reuse amongst hotel guests increases when the social norm for towel use is explicitly stated in the hotel room, e.g., [11,82,83]. For instance, Schultz et al. [83] found that a simple in-room message stating ‘Nearly 75% of hotel guests choose to reuse their towels each day’, increased towel reuse by 25%. Additionally, one of the most popular and successful interventions for reducing alcohol consumption amongst students, is providing written normative information of the amount of alcohol their peers consume [84]. This is based on findings that most students tend to try and match their drinking to their peers but overestimate how much they consume. On receiving information on how much their peers actually drink, they reduce their consumption [85]. Moreover, the inclusion of happy or sad faces on household energy bills to convey social approval or disapproval (i.e., injunctive social norms) of the amount consumed, has even been found to reduce energy consumption amongst those who previously had increased energy use [86].

Importantly, such normative messages (i.e., those that highlight the behaviour or expectations of most people) are found to be more effective than persuasive messages (e.g., those that tell people the impact of their behaviour) [87]. For instance, the inclusion of persuasive information about a product’s carbon footprint has had little success in increasing sustainable consumption [88]. This is particularly pertinent in the context of animal welfare, where behavioural change is often sought through the persuasive description of current farm animal production practices. Given findings that pro-environmental, pro-health and pro-sustainability behaviours can be enhanced by explicitly conveying information on normative behaviours, it is possible that the power of social norms can be harnessed in a similar way to align consumer action with their citizen animal welfare intentions [76,81,87].

As demonstrated above, most successful social norm interventions are based on telling people what others normally do or what others expect them to do in a given situation [24]. However, applying social norms to encourage pro-animal welfare behaviour requires careful consideration of where they are most likely to be effective. Dolan et al. [24] advise that one way to do this is to pick out a desirable norm that already exists, and then make it more salient by telling people about it. Napolitano et al. [26] (p. 554) frames the animal welfare citizen–consumer attitude–behaviour gap as a social norm issue:

“As citizens, [individuals] tend to approach socially sensitive topics, such as animal welfare, in a way that conforms to perceived social norms (i.e., showing a high sensitivity to animal welfare issues), whereas as consumers, individuals will often buy products obtained from systems criticised about their welfare standards, even when alternative products are available”.

Thus, even though a desirable social norm does exist, it does not appear to spill-over and positively influence consumer behaviour. As such, making this desirable norm more salient in a consumer context may help close the attitude–behaviour gap.

In this effort, the grocery store would appear the likely context. However, based on negative results from using normative messages to increase purchases of sustainably caught fish, social norm messages are argued to be ineffective in supermarkets because consumers are time-short and already have too many messages to process [89]. Yet, research has found that placing social norm ‘placards’ on shopping trolleys can increase the purchase of healthy foods (e.g., fruits and veg) [90]. As such, placing normative messages about animal welfare on shopping trolleys may be more effective than placing them on the already information-crowded product display shelves. Notably, Miller and Brannon [84] argue that tailoring a social norm intervention to an individual’s self-schema (e.g., personal values), will enhance its efficacy. This is because a tailored message is more likely to grab the individual’s attention and be critically, rather than peripherally, processed by the individual’s mental mechanisms. When, as posited above by Napolitano et al. [26], citizens already possess a sensitivity to animal welfare, then messages communicating the descriptive or injunctive norms of animal welfare at point-of-purchase may be effective, because they appeal to the consumer’s pre-existing values for animal welfare.

Online grocery shopping has also increased in recent years and provides a fruitful environment to nudge ‘animal-friendly’ purchases via social norms. In this regard, Demarque et al. [88] found that descriptive norms are effective incentive tools, or behavioural ‘nudges’, for encouraging ecological purchases in online shopping. When the online shop included a statement that “90% of previous participants purchased some ecological products”, participants spent 10% more of their money to place at least one ecological product in their basket. A significant finding of this study is that a strong norm message (e.g., 90% of people do this, as opposed to 20% of people do this) influences the behaviour of consumers who do not have pro-environmental attitudes. This is particularly pertinent for animal welfare, where consumers who do not have strong sensitivities to animal welfare are thought to be most sensitive to price [26]. Yet, as Demarque et al. [88] has shown, a strong descriptive norm message in an online shopping context can outweigh financial implications and prompt positive behaviours.

Telling people what not to do, what they do not do or what others are not doing, often reinforces or encourages undesired behaviours [91]. Within animal welfare, a lot of focus is placed on the public’s lack of knowledge of farm animal welfare and a lot of energy and resources go in to trying to increase this knowledge and inform them. Perhaps the lesson from social norms is rather than emphasising what citizens don’t know or what they are not doing, it is better to describe the positive things that they or others value. For example, a simple message stating ‘50% of people who buy free range eggs also buy higher welfare chicken’, may be more effective than saying not enough people are buying higher welfare products or using persuasive messages [87,88].

In sum, framing pro-animal welfare behaviours as something ‘others’ do and value [18] is a simple, yet powerful tool to potentially reduce the citizen–consumer attitude–behaviour gap. In designing such social norm interventions, it is critically important to emphasise desirable rather than undesirable norms [24] and to choose contexts where the desired behaviour is uncomplex and easy to preform [92]. As some positive social norms already exist amongst citizens (e.g., sensitivity to animal welfare) making these more salient in a consumer context is a beneficial starting point. Notably, behaviour change initiatives which take account of social norms spread rapidly because people take their cues from the behaviour of others [24]. 

### 3.4. Pre-Commitments

A close friend and ally of social norm interventions are pre-commitments. In a desire to maintain a positive reputation and consistent self-image, people try to be “as consistent as possible in their commitments, choices and behaviours… [as such] the need to be consistent with one’s commitments is a powerful influential tool” [11] (p. 11). Pre-commitments are a nudge tool which motivate people to commit to a goal which aligns their future behaviour with their desired self-image [24]. As pre-commitments require an individual to self-elect to place restrictions on, or change the incentives for, their future behaviour [93], it is an approach most suited to citizens who want to improve their pro-animal welfare consumer choices. 

Pre-commitments are most effective when the cost of failure is high. Committing publicly to a goal, choice or behaviour is one powerful way to increase the cost of failure, as reputational damage is risked when an individual shows a lack of consistency with public promises [24]. Furthermore, publicly stated attitudes are known to result in behaviours that are more consistent and stable over time [94]. Verbal commitments and promises have even been found to be more effective than economic penalties in reducing undesired behaviours [95]. A variety of studies have shown that publicly pre-committing to increase effort [95], lose weight [94] and get more exercise [96], increases the likelihood that an individual will stick to these goals because failing to do so results in social disapproval [24]. A ‘deposit contract’ is another type of pre-commitment, whereby volunteers deposit money into accounts they can only access on satisfactory completion of their goal [97]. This approach has been effective for getting people to quit smoking [98], take their medication and engage in other health-related behavioural changes [99]. However, with encouraging greater pro-animal welfare behaviour within society, the need is less to encourage people to commit to a long-term goal and more to encourage people to commit to a choice or behaviour. As such, public commitments seem the more appropriate form of pre-commitment devices to apply, particularly considering the previously discussed power of social norms in this context [27]. Furthermore, unlike the findings noted above where pre-commitments led to improvements to the health of an individual, a public commitment to animal welfare may have ‘warm-glow’ benefits; a phenomenon where individuals derive satisfaction from an act in and of itself, independent of its impact [100]. ‘Warm-glow’ is often linked to social norms, where the positive self-image ‘warm-glow’ creates is influenced by the extent to which an individual feels their behaviour is socially responsible [100]. As such, public pre-commitments seem the most relevant commitment device in the context of animal welfare.

Public commitment campaigns such as ‘Veganuary’, provide some insight into how pre-commitment devices could be used to enhance pro-animal welfare behaviours, particularly as animal-related reasons are often the primary motivation for following a vegan diet [101]. During the social media ‘Veganuary’ campaign, individuals are invited to make a public pre-commitment not to consume dairy for that month. Moreover, visitors to the ‘Veganuary’ website (veganuary.com) are prompted by a further pre-commitment tool to ’Take a pledge’, whereby they sign up to commit to living vegan for 31 days. The powerful and longer-term effect of such a commitment is evident in an article written by a UK veterinarian who notes that “Veganuary is over, and to my surprise, I find myself amongst the 51% of participants making a longer term commitment to a vegan lifestyle” [102]. The longer-time behaviour change impact of pre-commitment devices are well recognised, where a central effect of pre-commitments is the delay of short-term gratification (e.g., eat chocolate) to support a longer-term goal (e.g., eat healthily) (see Bryan et al. [103] for review). The longer short-term gratification is delayed, the greater the opportunity for behaviour change and habit formation to occur [104]. Hence the above noted success of a commitment to ’veganuary’ supporting habitual vegan choices. Indeed, it has been noted that such campaigns may have contributed to the increasing mainstream acceptance and rise of veganism [105]. 

The ‘veganuary’ campaign is quite an extreme and controversial example. However, it provides inspiration as to how pre-commitments could be used to encourage greater consumer commitment to purchasing higher welfare products. There is huge potential for public bodies, farm assurance schemes or other relevant groups to design pre-commitment devices encouraging consumers to commit to buying produce (e.g., meat, dairy, eggs) of a higher welfare standard for a specified period. Many already run marketing campaigns to inform consumers, so the addition of a pre-commitment device such as a public pledge, would be a simple, yet effective way to encourage higher welfare consumer behaviours. Once this commitment is made, additional educational material outlining how to buy such products and what labels to look for are likely to have greater impact. As discussed, a public pre-commitment device, potentially supported by social media platforms, is likely to be the most effective method [24,94] and can increase the likelihood of longer behavioural change and habit formation [104]. 

Pre-commitment devices work because they create accountability and they offset the self-control problems most decision-makers face [20] by increasing the cost of failure, either through the loss of a monetary incentive (e.g., with deposit contracts) or through social disapproval (e.g., with public promises). To work effectively, a pre-commitment device must both harness and emphasise a desired self-image (e.g., an animal welfare sensitive consumer) and include a penalty for not being consistent to that self-image (e.g., social disapproval). With some creative thought, there are many opportunities to apply pre-commitments in the context of animal welfare. As with many of the nudge tools described in this paper, such a behavioural intervention is favourable for its cost-effectiveness and parsimonious nature [24]. Furthermore, the long-lasting effect of successful pre-commitment devices on behaviour and their potential to support habit formation makes them an attractive tool for reducing the consumer attiude–behaviour gap affecting animal welfare. 

## 4. Discussion

Despite the rapid rise in the popularity of ‘nudging’ as a behaviour change tool, particularly amongst policy-makers and governments, it is not without its critics [12]. Many question whether such behaviour change techniques can be as effective as its proponents say [106] and whether nudging is even ethical [12,41]. As such, before implementing any nudge, it is important to consider its limitations, the conditions required for its effective implementation and the ethical implications of using nudges. As criticisms of nudging and its ethical implications are wide-ranging and varied, a full discussion is beyond the scope of this paper; however, many excellent and detailed critiques of nudging are available [41,75,106,107,108]. This section will focus on reviewing the limitations of the four nudges presented in this paper and provide a discussion of key ethical considerations.

Self-nudges rely on consumers having an awareness that they often digress from their intentions, and to work effectively, consumers must want to reduce such digressions and align their behaviours with their intentions [53]. However, when a central tenet of behavioural economics is that humans innately lack in self-control [22], it is not surprising that claiming self-nudges to be effective seems somewhat premature. Indeed, the empirical research into self-nudges is very limited, e.g., [46,107]. Torma et al. [53] found that self-nudges did improve long-term sustainable consumer behaviours (through organic food box subscription), while Wilson et al. [109] found that self-nudging did not improve long-term weight loss (amongst individuals with diabetes). Clearly, further empirical research is required to ascertain how and under what conditions self-nudges can be effective. Nevertheless, the key strength of self-nudges is that they are self-generated by the individual consumer. One of the issues brought against nudging generally, is that its effectiveness may be constrained by not being tailored to the individual and their self-schemas of the world [84]; individuals often diverge in responses due to differences in cultural, social or economic contexts [12,25]. Self-nudges overcome this issue, as it is the consumers themselves who decide how and when to change the choice architecture to nudge their behaviour [53]. Moreover, research has shown that the public prefer when the target of the nudge is an individual rather than wider society [110,111]. Furthermore, in being designed by the individual, self-nudges do not face the same ethical implications or criticisms of potential for coercion as choice architecture does (see discussion below). Thus, self-nudges may prove to be an effective behaviour change tool in the context of animal welfare and empirical research to examine this would be most welcome. However, it is important to keep in mind that the effectiveness of self-nudging is arguably limited to individuals who already possess a high motivation to improve their animal-welfare related consumer decisions.

Of all the nudge tools, choice architecture is perhaps the most well-known and analogous to the central premise of nudging; that changing the way in which information is conveyed in an environment can influence individual decision and behaviour. For this reason, choice architecture has received the most criticism, with many questioning the ethics of deliberately designing an environment to ‘take advantage of’ the innate biases of human behaviour without the individual’s awareness [41,75,107]. Bovens [108] even expresses concerns that choice architecture may hinder an individual’s development of moral character, where behaviour becomes fragmented between choice architect-designed environments and non-designed environments, thus reducing individual accountability for their own behaviour. For example, a consumer may buy higher welfare products in the supermarket (e.g., because of sectioning) but fail to make a higher welfare choice in a restaurant because similar choice architecture support is not there, thus limiting their personal development of moral accountability. An additional issue, as Selinger and Whyte [75] note, is that although the concept of choice architecture is straightforward, designing and applying them in practice can be difficult and confusing.

In response to such criticisms and ethical questions, Cass Sunstein, one of the seminal authors (along with Richard Thaler) on nudging and choice architecture, argues that:

“Both nudges and choice architecture are inevitable, and it is therefore pointless to wish them away…many forms of choice architectures are defensible and even required on ethical grounds, whether we care about welfare, autonomy, dignity, self-government, fair distribution, or some other value”.

[47] (p. 4)

What he means by this is that every decision a person makes is done against a background of choice architecture; every choice environment has a design and that design affects choice. As such, the critical argument for the use of choice architecture is that it is better to deliberately design this environment in a way that supports the welfare or best interests of individuals and society, rather than leaving it to chance [47]. A critical ‘litmus test’ then for choice architecture is that it must not limit any individual’s decisions; it can suggest but not coerce [9]. The sectioning of animal welfare products, as described in this paper, arguably passes this as individuals can still choose amongst other alternatives but those who are attracted to the higher welfare section are less hindered by reference pricing biases in their choices. Sunstein [40] further notes that any nudge tool must be transparent, not hidden or covert. Sectioning of animal welfare products adheres to this, as it is very visible to consumers that a change has been made to the environment. However, consumers will not know the reason for this change, hence the ethical and moral question mark fueling the on-going debate about choice architecture [41]. It is for this reason that Sunstein [40] cautions that before any choice architecture (or other nudge tool) intervention is rolled out, it is essential that it has been tested empirically, preferably through randomised controlled trials. Although changes to supermarket choice architecture have been tested empirically in other contexts, animal welfare would represent a new departure for choice architecture and needs empirical research to test the suggestions made in this paper.

The communication of social norms, both descriptive and injunctive, is a behaviour change tool with a rich empirical research base [76,78,112,113]. As such, there is a substantial body of research depicting when, and under what conditions, social norm interventions do and do not achieve desired results. One notable issue with social norms is the boomerang effect, when social norm messages have resulted in a decline in the target behaviour compared to the baseline [89]. In trying to understand why this occurs, Richter et al. [89] speculated that consumers may have perceived the social norm message as too insistent, invasive or constraining of their personal freedom of choice, and thus resisted it [89]. This highlights a potential ethical implication of social norms; individuals may feel it limits their choice or is pushing them to engage in a particular behaviour. Proponents of nudging would argue social norms serve as a hint to desired behaviour and consumers can choose to ignore the message. However, findings such as those of Richter et al. [89] suggest otherwise and highlights how the choice-preserving tenet, central to nudging, must not be ‘taken-for-granted’ but deliberately considered in its design [75]. Moreover, it is important to consider the ethics of the social norm message itself. Previous studies have used an ‘untrue’ (e.g., not based on a factual or accurate statistic) message and found it can positively affect consumer behaviour [16,88]. Yet, is it acceptable to tell an untruth even if it improves a person’s welfare (e.g., their health, supports pro-animal welfare intentions)? [16]. For most researchers, this is morally and ethically unacceptable but others may argue that the ends justify the means [16].

A further limitation of social norms is that some consumers may not fully process or notice the social norm message, “as in-store decisions are characterised by time pressure and the use of simple heuristics, expecting consumers to read a relatively long text might not be realistic in this type of environment” [89] (p. 10). As such, supermarket on-shelf or on-counter social norm messages are arguably not the best conditions for an effective social norm intervention. An additional issue is that because social norms tend to communicate the average behaviour of a group, those who normally behave below the average may then change behaviour to conform to the average. This has been particularly problematic in energy consumption interventions, where citizens with below average consumption increased to that of the average, causing a worsening in their energy consumption behaviour [114,115]. Thus, it is very important to consider what exactly a social norm message is conveying [24]. Melnyk et al.’s [113] meta-analysis of social norms in consumer behaviour gives some insight as to how and when social norms work best. Namely, social norm message are most effective when they come from concrete (e.g., authority figure) sources and when everyday consumption choices, such as food, are the target, rather than more complex choices. Such findings emphasise the importance of close consideration of what message is conveyed and to whom it is delivered; a clear understanding of how, when and to whom are critical to the effective design of social norm interventions [24].

The use of pre-commitments to overcome self-control problems (e.g., wanting to choose pro-animal welfare products but failing to) has a strong empirical base [24,94,99]. However, considering people lack in self-control, a critical question is whether individuals have the self-control to self-impose and adhere to meaningful commitments. This is a question examined by Ariely and Wertenbroch [116] in the context of student performance. They conclude that, yes, individuals are willing to pre-commit to a behaviour or a goal where failure to adhere to them can incur penalties (e.g., social or monetary). Interestingly, however, Munson et al. [96] find that public pre-commitments may actually decrease the probability an individual will make a commitment in the first place, as they fear the negative attention they may receive if they fail. Thus, pre-commitments, even though individuals self-select to use them, are not without their limitations or ethical considerations. Indeed, Selinger and Whyte [75] questions whether anything which imposes a penalty—as public pre-commitments do through the penalty of social disapproval—on an individual can be considered a nudge, as they argue it works more by changing the incentive of a behaviour than by influencing a behavioural bias (e.g., self-control issues). Such criticism is reflective of the wider debate surrounding nudging, where its proponents argue it can be used to help people fulfill their goals and intentions, while its critics say it unnecessarily limits individual free-will and hinders personal accountability [41,108].

Overall, the primary ethical issue associated with nudging is that the very interventions which can be used to help consumers align their actions with their intentions, may be misused to manipulate them [16]. Arguably, the key issue here is the extent to which nudges restrict consumer free will or autonomy. In this regard, Baldwin [12] typifies three levels of nudging, each differing in the degree to which they affect individual autonomy: ‘first-degree’ nudges fully allow autonomy and encourage reflective decision-making (e.g., self-nudges); ‘second-degree’ nudges place some limitations to encourage choice in a desired direction (e.g., choice architecture, social norms and pre-commitments); and ‘third-degree’ nudges limit choices and obstruct individual reflection on how the intervention has influenced them [12]. As with any behavioural intervention, nudging is not without its limitations and its use requires very careful consideration. As Ratner et al. [16] (p. 393) highlight, “researchers, who design interventions and policies to help consumers, may confront these ethical controversies (or debates) that have no simple answers and that sometimes force researchers to consider trade-offs between multiple ethical principles they hold”. How, when and whether nudging is ethical is an on-going debate which becomes ever more prevalent with the increasing popularity of such interventions [75,107,108]. In the absence of a strict code or protocol for their application, it is essential that researchers or policy-makers interested in applying nudges consider the ethical implications of their use carefully before they are applied.

## 5. Conclusions

As the market-driven nature of animal welfare continues to rise in many countries, encouraging consumers to engage in greater pro-animal welfare behaviours is arguably of importance. However, despite the increasing availability of higher-welfare products and the palpable rise of interest in farm animal welfare, demand for ‘animal-friendly’ products does not appear to reflect the stated desire for them [4,26]. If people are expressing pro-animal welfare attitudes but not pro-animal welfare behaviours, then perhaps, they are not being properly supported to make the decisions they want to. In response, this paper drew from behavioural ‘nudge’ interventions applied in fields (e.g., health [99] and the environment [11]) which face similar problems to that of farm animal welfare; a clear gap between citizen intention and consumer action. In this effort, this paper has sought to introduce to animal welfare scientists the nuance and complexities affecting human behaviour, particularly how it is driven by unconscious automatic processes which are highly susceptible to the design of the context, how information is presented, the behaviour of others and mental shortcuts. Recognising that our behaviour can be influenced by the way in which information is presented means we can design suitable choice architecture to ‘nudge’ desired behaviours or indeed, ‘self-nudge’ ourselves. Similarly, knowing that many of our decisions are driven by a need to adhere to social norms means we can affect behaviours by explicitly describing positive and desirable norms. Finally, the knowledge that self-control issues can be overcome through pre-commitment devices enables us to develop tools to effect longer term behaviour change and support ‘higher welfare’ as a habitual choice. Designing behavioural change initiatives with these in mind may serve to better align citizens’ pro-animal welfare intentions with their consumer action.

The behavioural initiatives introduced in this paper are by no means a definitive list of ‘nudges’ or behavioural economic tools. Rather, they were chosen for their potential applicability to reducing the citizen–consumer attitude–behaviour gap. Each of the nudges presented in this paper are somewhat interconnected and can reinforce one another. For instance, self-nudges work by making salient to an individual the desire to be consistent with their self-image when buying products. Pre-commitments work similarly by harnessing the desire to be consistent with an individual’s self-image, but further reinforce this by imposing a penalty for lack of consistency. Social norm interventions further contribute to this by communicating what others do or what others consider acceptable, in order to nudge individuals into making positive choices. Finally, choice architecture supports decision-making at critical times by ensuring that the way in which information is presented supports, rather than hinders, ‘animal-friendly’ decisions. As Sunstein [40] notes, an important goal for any ‘nudge’ is to ‘increase navigability’, or make it easier for people to carry out their desired intentions. Considering the citizen–consumer attitude–behaviour gap in animal welfare, this is a worthy and beneficial goal for nudge interventions in this context. If people have a desire to make ‘animal-friendly’ decisions, then the environment in which those decisions are made should support them to do so; this is the central argument for ‘nudging’.

This paper was intended as a mere introduction to some of the ways in which nudge interventions could enhance consumer behaviour in relation to farm animal welfare. The examples of how nudges could be applied to animal welfare, although grounded in empirical findings, are purely hypothetical and are included to illustrate where they could be applied, how they work, and why they may be effective. It is hoped that others will be inspired to think about how they could be applied to other areas where behavioural change relevant to animal welfare is needed and, indeed, to critique the examples suggested here.

## Figures and Tables

**Table 1:** Description of four nudge tools, with examples of application to animal welfare and description of the behavioural mechanisms harnessed by each nudge.

‘Nudge’	Description	Example	Behavioural Mechanisms
**Self-nudges**	Techniques individuals use to ‘nudge’ themselves to behave according to their desired intentions, often by avoiding decision contexts where desired choice is difficult or altering their environment so desired choices are easier to make.	Consumer signing up to a ‘higher welfare’ meat home delivery service.	Avoids the hyper-choice complexity of the supermarket, where systems 1 (our fast, intuitive thinking brain can influence impulsive decisions). Makes salient self-image as an ethical consumer, allowing reflection in systems 2 thinking and reinforcing future purchases.
**Choice Architecture**	The deliberate altering of the choice environment, by a ‘choice architect’ to better harness or overcome the behavioural biases influencing choice in the target context, without limiting alternative choices.	Partitioning the supermarket so that higher welfare products are in their own specific section or aisle.	Reference points (e.g., decisions are made by evaluating choices relative to reference price). Information salience (e.g., less cognitive effort required to uncover ‘higher welfare’). Choice reduction (e.g., reducing cognitive overload when making decision).
**Social Norms**	The implicit rules and expectations of a social group which guide and influence behaviour, where individuals seek to adhere to what is socially acceptable even if this deviates from their own self-interests.	Message describing what most others who purchase a product (i.e., descriptive social norm) do in an online shopping environment, e.g., “50% of people who buy free range eggs also buy higher welfare chicken breasts”.	Unconscious ‘follow-the-crowd’ effect. Desire to avoid social disapproval. Look to decisions of others when facing complex choices or uncertainty.
**Pre-commitment devices**	In recognition of short-term self-control issues, individuals make a commitment to achieve a long-term goal or engage in a specific behaviour. Most effective when the cost of failure is high.	Encouraging consumers to publicly commit (e.g., through social media) to purchase higher welfare products for a stated period.	Desire to be consistent with public image. Wanting to be consistent with desired self-image
